# Editorial: The Updated Understanding of Stevens-Johnson Syndrome and Toxic Epidermal Necrolysis

**DOI:** 10.3389/fmed.2021.811570

**Published:** 2021-12-22

**Authors:** Shigeru Kinoshita, Mayumi Ueta

**Affiliations:** ^1^Department of Frontier Medical Science and Technology for Ophthalmology, Kyoto Prefectural University of Medicine, Kyoto, Japan; ^2^Department of Ophthalmology, Kyoto Prefectural University of Medicine, Kyoto, Japan

**Keywords:** Stevens-Johnson Syndrome (SJS), toxic epidermal necrolysis (TEN), severe ocular complications (SOC), human leukocyte antigen (HLA), international collaboration

Stevens-Johnson Syndrome (SJS) is an acute inflammatory vesiculobullous reaction of the skin and mucosa, including the ocular surface, oral cavity, and genitals. In SJS patients with extensive skin detachment and a poor prognosis, the condition is called toxic epidermal necrolysis (TEN). The treatment of both SJS and TEN is extremely difficult, and in fact, in our Ophthalmology Residency Program in the 1970s, we were instructed that there was currently no surgical or medical treatment for visual disturbance of patients afflicted with SJS/TEN. However, for corneal specialists, proper treatment of the devastating SJS/TEN-associated ocular surface disorder is vital. Depending on the drugs and infections involved, SJS/TEN is not always accompanied by severe ocular complications (SOC). However, the subgroup of SJS/TEN develops SOC due to corneal epithelial stem cell deficiency at the acute phase, thus resulting in severe visual impairment that prolongs into the chronic phase as well. The corneal opacity in SJS/TEN cases cannot be treated with regular corneal transplantation due to the ocular-surface stem cell deficiency and the proliferative changes caused by postoperative inflammation. Thus, when in search of a treatment for such devastating ocular surface disorders that cannot be treated with regular corneal transplantation, the use of several surgical strategies, such as keratoprosthesis (1), have been tried. In 2002, our team developed a new treatment method for cases with severe ocular surface disorders, known as Cultivated Oral Mucosal Epithelial Transplantation (COMET) (2). In SJS/TEN patients with severe visual impairment, treatment with COMET might provide a novel pathway toward the improvement of vision and overall quality of life. Recently, we developed a limbal-supported hard contact lens for use after the COMET procedure (3), which we found improved the patient's visual acuity. Hence, patients with SJS/TEN (which is an extremely rare disease) with SOC are now visiting the Department of Ophthalmology at Kyoto Prefectural University Hospital from all over Japan for treatment.

Previously, research on SJS/TEN was primarily conducted by dermatologists, as they are the clinical specialists who treat SJS/TEN patients in the acute phase in many countries, including Japan, Taiwan, Korea, and Europe. However, ophthalmologists also treated chronic SJS/TEN patients with ocular sequelae, such as the visual impairment that exists throughout their lives. Hence, we ophthalmologists have now joined with dermatologists in a nationwide Japanese SJS/TEN Study Group to deepen our understanding on SJS/TEN patients with and without SOC, complications that can lead to ocular sequelae in the chronic phase. Quite surprisingly, this partnership has allowed us to more deeply understand SJS/TEN-related ocular sequelae, as well as the overall condition and quality-of-life setbacks that SJS/TEN patients endure. Moreover, we discovered that not all SJS/TEN patients are afflicted with SOCs, such as severe conjunctivitis with ocular-surface persistent epithelial defects and pseudomembrane formation in the acute phase. To that regard, in collaboration with the Japanese Research Committee on Severe Cutaneous Adverse Reaction, our findings revealed that ~80% of all SJS/TEN patients had experienced conjunctivitis, and that ~50% of all SJS/TEN patients have experienced severe conjunctivitis with ocular surface epithelial defects and/or pseudomembrane formation (4).

A dermatological study revealed that anticonvulsants (i.e., carbamazepine, etc.) and anti-gout drugs like allopurinol were critical causative agents for SJS/TEN (5). However, when we performed detailed interviews with SJS/TEN patients with SOC who attended our hospital about their condition at the onset of SJS/TEN, we found that only about 5% of those patients had developed SJS/TEN after taking anticonvulsants, and that almost none of the patients had developed SJS/TEN after taking allopurinol. Moreover, our human leukocyte antigen (HLA) typing analysis findings of Japanese SJS/TEN patients with SOC, which showed that HLA-A^*^02:06 and HLA-B^*^44:03 was significantly associated with those complication (6), were also completely different from the HLA types reported in the above-mentioned dermatological study; i.e., carbamazepine-induced SJS/TEN manifested a very strong association with the HLA-B^*^15:02 allele in Taiwanese Han Chinese patients (7), and with the HLA-A^*^31:01 allele in Japanese (8) and European patients (9), and that allopurinol-induced SJS/TEN was strongly associated with HLA-B^*^ *58:01* in Han Chinese (10), Caucasian (11), and Japanese patients (12). Hence, we have now come to realize that SJS/TEN patients with SOCs might actually constitute a subgroup of all SJS/TEN patients treated by dermatologists, and have focused our attention on SJS/TEN patients with SOC at the acute phase and ocular sequelae at the chronic phase by investigating the epidemiology, causative agents, and associated pathogenesis.

Surprisingly, in 2009 we discovered that the average age at disease onset of the SJS/TEN patients with ocular sequelae treated at our hospital was 26 years (13), which is about 20 years younger than the average age at disease onset reported by dermatologists in 2007 (14). Moreover, disease onset in ~80% of our SJS/TEN patients with SOC occurred after taking cold medicine, such as multi-ingredient medications including acetaminophen and non-steroidal anti-inflammatory drugs (NSAIDs), thus indicating that the causative drugs for SJS/TEN with SOC might be unique. Furthermore, in SJS/TEN patients in which the disease is caused by antiepileptic drugs or allopurinol, onset can occur 2 weeks after administration of the causative drug. In cold medicine-related SJS/TEN with SOC, onset can occur within 1 week after taking the medication. Thus, we now theorize that SJS/TEN with SOC might be a subgroup of the overall SJS/TEN population. However, it has been challenging to obtain an international consensus of our concept, because the world's leading dermatologists on SJS/TEN have yet to embrace of finding that cold medicine can cause SJS/TEN. Moreover, a leading dermatological group has argued that the onset of SJS/TEN within a few days after the administration of cold medicine was more likely due to an infection, and not the medication (5).

Hence, we subsequently developed an international collaboration with ophthalmologists who have treated SJS/TEN patients with SOC in order to demonstrate that our concept is both appropriate and acceptable. Fortunately, the COMET procedure developed by our group has now been used to some extent worldwide, and many ophthalmologists now perform the procedure for the treatment of SJS/TEN with SOC. Initially, we began collaboration on causative agents and genetic predisposition of SJS/TEN with SOC in Korea, followed by Brazil and India. As we proceeded with these international collaborations, we were selected for the International Research Center Formation Project Core-to-Core Program A; Advanced Research Networks, funded by JSPS (the Japan Society for the Promotion of Science). Using this research funding, we have now expanded our international collaborations and have been holding the annual “International Stevens-Johnson Syndrome Symposium” in Japan since 2016. At present, in addition to Korea, Brazil, and India, we collaborate on this project with Thailand, Taiwan, the United Kingdom, the United States, and Singapore. Moreover, we not only collaborate with ophthalmologists, but also basic scientists in the field of genomic analysis and immunologists to elucidate the genetic predisposition and pathogenesis of SJS/TEN. We also hold discussions with dermatologists to further elucidate the difference between SJS/TEN with SOCs and without SOCs.

In this way, the International Stevens-Johnson Syndrome Symposium has now brought together not only ophthalmologists and dermatologists, but also epidemiologists, genomic researchers, immunologists, and researchers from other medical fields. We discuss the advanced current clinical treatment, epidemiology, genetic predisposition, and pathogenesis of ophthalmic SJS/TEN, which now greatly enhances our knowledge and improves the level of understanding about ophthalmic SJS/TEN worldwide.

In conclusion, this special issue consists of many mini-reviews from our collaborators showing their advanced research results; i.e., the clinical aspect of SJS/TEN with SOCs in each country (i.e., in Japan, Korea, Taiwan, Thailand, Singapore, India, the United States, and the United Kingdom), from the pathological and genomic aspects of SJS/TEN with SOC. It is our hope that this special issue will raise the level of understanding of SJS/TEN throughout the world, and we hope that it will contribute to the further elucidation of the pathogenesis of ophthalmic SJS/TEN, including its genetic predisposition, and the future development of novel treatment modalities.

## Author Contributions

All authors listed have made a substantial, direct, and intellectual contribution to the work and approved it for publication.

## Funding

This work was supported by grants-in-aid from the Ministry of Education, Culture, Sports, Science, and Technology of the Japanese government, by the JSPS Core-to-Core Program, A. Advanced Research Networks, and partly supported by grants-in-aid for scientific research from the Japanese Ministry of Health, Labor, and Welfare.

## Conflict of Interest

The authors declare that the research was conducted in the absence of any commercial or financial relationships that could be construed as a potential conflict of interest.

## Publisher's Note

All claims expressed in this article are solely those of the authors and do not necessarily represent those of their affiliated organizations, or those of the publisher, the editors and the reviewers. Any product that may be evaluated in this article, or claim that may be made by its manufacturer, is not guaranteed or endorsed by the publisher.
